# The Immunity Gap Challenge: Protection against a Recent Florida Clade 2 Equine Influenza Strain

**DOI:** 10.3390/vaccines6030038

**Published:** 2018-07-02

**Authors:** Romain Paillot, Dion Garrett, Maria R. Lopez-Alvarez, Ihlan Birand, Fernando Montesso, Linda Horspool

**Affiliations:** 1 Animal Health Trust, Lanwades Park, Kentford, Newmarket CB8 7UU, UK; diongarrett013@gmail.com (D.G.); MARIA.LOPEZ@aht.org.uk (M.R.L.-A.); ilhanbirand@hotmail.com (I.B.); fmontesso66@gmail.com (F.M.); 2 LABÉO Frank Duncombe, 14280 Saint-Contest, France; 3 Laboratoire BioTARGen, Université de Caen Normandie, 14280 Saint-Contest, France; 4 MSD Animal Health, 5830 AA Boxmeer, The Netherlands; linda.horspool@merck.com

**Keywords:** equine influenza, horse, vaccination, duration of immunity

## Abstract

Vaccination is one of the most effective tools for limiting the impact of equine influenza (EI). The humoral immunity established following a primary vaccination course can decrease significantly between the second (V2) and third immunisations (V3), leaving some horses insufficiently protected for several weeks. This so-called “immunity gap” poses a challenge to all EI vaccines. During this period, the EI infection of vaccinated animals may be followed by marked clinical signs and virus shedding. However, several EI vaccines have been shown to stimulate equine influenza virus (EIV)-specific cell-mediated immunity, which is likely to play a role in protection against EIV infection and/or mitigate the clinical and virological signs of EI. Reducing the interval between V2 and V3 has been shown to be counterproductive to longer-term immunity. Further research is needed to define and address the “immunity gap” in horses. This study aimed to measure the level of protection induced by a whole inactivated, ISCOMatrix adjuvanted, EI and tetanus vaccine (Equilis Prequenza-Te) when challenged during the immunity gap (i.e., immediately before the recommended boost immunisation, more than 5 months after V2) using infection with a recent heterologous Florida Clade 2 (FC2) equine influenza virus (EIV) strain. This vaccine was tested in a Welsh mountain pony model. A group of seven ponies was vaccinated twice, 4 weeks apart. The protective antibody response was measured and ponies were challenged, along with 5 unvaccinated control ponies, by experimental infection with the FC2 A/eq/Northamptonshire/1/13 EIV strain, 158 days (around 5.2 months) after V2 and their clinical signs and virus shedding were monitored. EI serology was measured by single radial haemolysis (SRH) and haemagglutination inhibition (HI). Clinical signs and virus shedding (measured by qRT-PCR and hen’s egg titration) were compared with controls. All vaccinates had detectable**,** low SRH antibody titres and most had detectable, low HI titres. Significant clinical and virological protection was observed in vaccinates (*p* < 0.05), supporting the good performance of this vaccine against a recent EIV strain. In this study, the impact of the immunity gap in ponies was limited after primary vaccination with this whole inactivated, ISCOMatrix adjuvanted EI and tetanus vaccine (Equilis Prequenza-Te) when infected several months after V2 with a recent FC2 strain, which is representative of EIV circulating in the EU.

## 1. Introduction

Equine influenza (EI) is a major viral respiratory disease in horses associated with high morbidity and potential catastrophic economic consequences [1]. To date, vaccination remains one of the most effective methods of preventing equine influenza virus (EIV) infection (reviewed in [2]). Most commercially available EI vaccines require a vaccination schedule that consists of two primary immunizations (V1 and V2), usually 4 to 6 weeks apart, followed by a boost immunization (V3), 5 to 6 months after V2. Further boost vaccinations are usually administered every 6 months or 1 year, depending of the vaccine used, the local veterinary vaccination practices, regulations, requirements and the risk of exposure to EIV. 

The efficacy of vaccines against EIV has been widely demonstrated and documented, both in experimental infection studies in the natural host (reviewed in [2]) and/or during outbreaks following vaccine use under field conditions [3,4,5,6,7]. The EI vaccines available commercially reduce the clinical signs and virus shedding following EIV infection and do not generally produce long-lasting sterilising immunity (i.e., protection against infection beyond what is demonstrated at or around onset of immunity (2–3 weeks after V2)). Such protection is primarily correlated with the level of circulating antibodies to EIV, with the single radial haemolysis (SRH) and haemagglutination inhibition (HI) assays being suitable for measuring EIV haemagglutinin (HA)-specific antibody titres in horses [8]. It is also important to note that most EI vaccines have been shown to stimulate EIV-specific cell-mediated immunity [2], which is likely to play a role in protection against EIV infection and/or in the mitigation of clinical signs and virus shedding following EIV infection. Following vaccination, antibody titres measured by SRH have been shown to correlate well with protection against infection following experimental or field infection. Thresholds have been defined previously for SRH antibody titres that correlate positively with a reduction in clinical signs of EI (titres > 85 mm^2^) and virus shedding (titres > 120–154 mm^2^) after infection with an EIV strain that is homologous to the virus strain contained in the vaccine [9,10]. This is supported by more recent experimental EI vaccine studies and field data [2,6].

The peaks of protective humoral immunity to EIV are usually measured 2–3 weeks after V2 and subsequent immunisations (V3, V4, etc.). However, EIV HA-specific antibody titres established following a primary vaccination course have been shown to decrease significantly between V2 and V3 [11,12], leaving some horses insufficiently protected and with increased susceptibility to EIV infection for several weeks. This period of susceptibility is the so-called “immunity gap” [13] and poses a challenge to all EI vaccines. The risk and consequences posed by the immunity gap are not insignificant. If vaccinated horses are infected with EIV during this period, they may develop little (mild) or no (subclinical) clinical signs of EI but may shed increased amounts of the virus. In this condition, the risk posed to other horses, particularly naïve (unvaccinated) horses, could be significant. This has been confirmed in recent studies [14,15], which showed that vaccinated horses (with a vaccine containing H7N7 and H3N8 American and European lineages or H3N8 European lineage, respectively) experimentally infected with EIV (A/equine/Richmond/1/07 Florida clade 2 sublineage or /Newmarket/1/1993 American lineage, respectively) 78 days (i.e., around 2–3 months) after V2 and/or when SRH antibody titres were mostly below 60 mm^2^ could shed live infectious EIV for several days and infect naïve sentinels. Such a risk was also clearly illustrated during the devastating 2007 Australian EI outbreak, which was likely triggered by the importation from Japan of horses with subclinical EIV infection [16]. According to their vaccination records, all horses had received a primary immunisation course (V1 and V2) prior to their export to Australia. The horses were shown to have seroconverted to EIV between the pre-export and post-arrival quarantine samples, tending to indicate that infection occurred 2 to 4 months after V2 (i.e., during the immunity gap). More recently, the epidemiological analysis of EI outbreaks in Ireland between 2010 and 2012 showed that only one-third of premises affected had up-to-date vaccination records for less than one-half of the horses present and that several vaccinated horses (vaccines contained H7N7 and H3N8 American and European lineage strains but did not contain Florida sublineage Clade 1 or 2 strains) had been infected with EIV during the immunity gap (i.e., 2 to 5 months after V2) [6]. 

Several attempts to “close” the immunity gap have been reported. For example, a reduced interval between V2 and V3 (e.g., 8 weeks post V2) led to lower antibody titres compared to horses administered V3 according to the manufacturers recommendations (i.e., 24 weeks after V2) [17]. High titres of EIV HA-specific antibody at the time of V3 may have hampered the anamnestic response induced by boost immunisation (e.g., interference of pre-existing EI antibody response with the EI vaccine administered) [11]. Overall, most of the evidence available indicates that product label recommendations should be followed as far as possible, with little room available to reduce the interval between V2 and V3 without impacting mid- and longer-term protection. In this context, the evaluation of protection during the immunity gap is important to assess the actual risk to vaccinated horses and in contact horses within a herd.

The study reported here aimed to measure the level of protection induced by a whole inactivated, ISCOMatrix adjuvanted, EI and tetanus vaccine when challenged at the peak of the immunity gap (i.e., immediately before the recommended boost immunisation, more than 5 months after V2 in the current study) using infection with a recent Florida Clade 2 (FC2) EIV strain (A/equine/Northamptonshire/1/13) (Table 1).

## 2. Materials and Methods

### 2.1. Experimental Animals and Vaccination Protocol

Eligibility criteria: All 12 Welsh mountain ponies (10 males and 2 females, approximately 11 months of age) demonstrated a good health condition and were seronegative for EIV, with no history of exposure to EIV or vaccination against EIV at the time of the study.

Setting, location and sample size: Ponies were enrolled by the study investigator. Twelve ponies were used and housed together at the AHT premises. During the infectious period of the study (from 2 days prior to experimental infection with EIV to 14 days after infection), the 12 ponies included in the study were assigned to 2 rooms of the category II containment facility, with 5 and 7 animals per room, respectively. Sample size (5 control ponies and 7 vaccinated ponies) was based on the European Pharmacopoeia criteria for EI vaccines (inactivated), which requires no fewer than 6 and 4 horses for the treated and control groups, respectively [18].

Investigational veterinary product (IVP) and intervention: The vaccine Equilis Prequenza Te (MSD Animal Health, 5830 AA Boxmeer, The Netherlands IVP; lot A207B01, expiry date October 2015, stored at 2 to 8 °C until use) was tested in this study. This vaccine contains the whole inactivated EIV strains A/equine/South Africa/4/03 (H3N8, American lineage, Florida Clade 1 sublineage, 50 antigenic units) and A/equine/Newmarket/2/93 (H3N8, European lineage, 50 antigenic units), tetanus toxoid (40 Lf), and is ISCOMatrix adjuvanted (purified saponin, cholesterol and phosphatidyl choline). Control ponies received a negative control/placebo (CP) (phosphate buffered saline (PBS), reference 10010-015 500 mL, lot: 1,666,825, expiry date: March 2017, stored at room temperature). The IVP and CP were administered by deep intramuscular injection (21G × 1½″, 0.8 × 40 mm needle) in the left neck (1 dose = 1 mL) on days 0 and 28. Vaccination took place within the stated shelf-life of the IVP/CP. On days 61 and 62, 10 male ponies were castrated. All ponies enrolled in the study were administered 10 mL (10,000 IU) of anti-tetanus serum (Tetanus antitoxin Behring from MSD Animal Health).

Randomisation and masking**:** The 12 ponies were assigned to 2 groups (control and vaccinated groups) of 5 and 7 animals, respectively. An online randomisation generator (www.randomization.com) was used with 2 treatment labels (Groups 1 and 2) and with 14 as the number of subjects per block and 1 as the number of blocks. Two subjects (Group 2) were removed from the generated list in order to obtain a randomisation of 12 subjects, with 5 subjects in Group 1, and 7 subjects in Group 2. The randomisation list (1 to 14, with 2 subjects/numbers in Group 1 removed) was renumbered from 1 to 12 in order to correspond to the list of ponies (*n* = 12).

Each group was numbered A1 to A5 for Group 1 and B1 to B8 for Group 2 (organised by increasing arithmetic ID). The online randomisation generator (www.randomization.com) was used, with 2 treatment labels (room 1 and 2), 6 as the number of subjects per block and 1 as the number of blocks. This procedure was repeated for Group 2 ponies, with 8 as the number of subjects per block and 1 as the number of blocks. Room #1 contained 5 ponies (2 controls and 3 vaccinates) and room #2 contained 7 ponies (3 controls and 4 vaccinates). The unequal pony allocation to the containment facility rooms was motivated by the diagnosis of an early case of Sweeney disease/shoulder in one of the ponies, shortly before infection. After veterinary examination, this pony was deemed fit to be experimentally infected with EIV, but the number of ponies per room was altered to provide this pony with a little bit more room (i.e., allocated to room #1, with only 4 other ponies) to minimize any possible discomfort.

Personnel at the study site responsible for performing clinical and general health observations or involved in laboratory assays were not informed about the allocation of individual animals at any time during the study.

Animal welfare: This study was carried out in compliance with the UK Animals Scientific Procedures Act 1986, with the approval of the Animal Health Trust (AHT) and MSD Animal Health Ethics Review Committees. All procedures were conducted at the AHT. This report of clinical trials follows the CONSORT 2010 guidelines (CONSORT Supplementary Materials Table S1 and flow chart) [19,20].

### 2.2. Viruses and Experimental Infection with EIV

Viruses were all grown in embryonated hens’ eggs, purified and titrated as described previously [21]. The EIV strain A/equine/Northamptonshire/1/13 (H3N8, Florida Clade 2 sublineage EIV strain, passage 3 in hens’ eggs, obtained from the AHT/Horserace Betting Levy Board EI UK surveillance scheme [7]) was used for experimental infection and was titrated in embryonated hens’ eggs before infection (day 186). The titre was defined using the method of Reed and Muench and expressed in logEID_50_ per mL [22]. The ponies were experimentally infected by individual nebulisation 1.5 mL of an infectious EIV suspension containing a total of 10^7.24^ EID_50_ EIV strain A/equine/Northamptonshire/1/13 (Flexineb^®^ 2, Progress Equine Ltd., Hungerford, UK) per animal [23]. The EIV strains A/equine/South Africa/4/03, A/equine/Richmond/1/07 (H3N8, Florida Clade 2 sublineage EIV strain) and A/equine/Northamptonshire/1/13 were used as SRH and HI antigens.

### 2.3. Serology

Outcome measurement: Serum samples were collected on day-1 (V1-1 day), day 7, 14, 21, 28 (V2), 35, 42, 56, 69, 82, 110, 148, 186 (experimental infection), day 193 and day 200, and analysed by SRH and HI assays [8] against the EIV strains A/equine/South Africa/4/03, A/equine/Richmond/1/07 and A/equine/Northamptonshire/1/13 used as antigens. SRH antibody titres were expressed as the area of haemolysis (mm^2^). An increase of at least 25 mm^2^ or 50% in the area of the zone of haemolysis was regarded as significant. European Pharmacopoeia reference serum standards EU SA/4/03 Y0000712 (A/equine/South Africa/4/2003) was used as a positive control sera (data not shown). HI antibody titres were expressed as the last dilution that inhibits chicken red blood cells haemagglutination in presence of EIV, as previously described [24]. A titre >8 was regarded as significant and a two-fold increase of the titre indicative of seroconversion. For statistical and graphical reasons, a HI titre <8 was converted into 4. Values for internal reference serum standards used during the assay were within the expected range. Serum samples were collected from all ponies on days-1 (V1-1 day) and 56, 69 and 186 (experimental infection) and analysed for tetanus toxoid-binding (ToBI) antibody titre as previously described [25]. Tetanus toxoid (TT) antibody titres were expressed as international units (IU)/mL of serum. 

### 2.4. Clinical Signs of Disease (Pre-Specified Analysis)

Outcome measurement: Clinical examinations were performed daily on each pony from day 184 (2 days before infection) until day 200 (14 days after infection) for the occurrence of clinical signs associated with EI as described previously [12,26]. Rectal temperature greater than 38.8 °C was regarded as pyrexia. The total clinical score after experimental infection with EIV was calculated using a daily score for each clinical sign according to the formula used at the AHT and reported previously [26]:

sickness score = (2 * score RT) + score nasal discharge + score cough + 2 * (score dyspnoea + anorexia + depression)

Ponies that developed moderate to severe and long-lasting (>3 days) clinical signs after experimental infection with EIV were treated with potentiated sulphonamide antibiotics (trimethoprim/sulfadiazine, TMPS) for 7 days to control secondary bacterial infection and improve recovery, as per the ethical guidelines of the AHT.

### 2.5. Virus Shedding

Outcome measurement: nasopharyngeal swabs were taken from each pony one day before infection (day 185) and daily for 14 days from days 187 to 200, with the exception of day 186 (experimental infection). Swabs were processed in 5 mL of virus transport medium (PBS, 200 U/mL streptomycin, 150 U/mL penicillin, 5 mg/mL amphotericin B and 600 mg/mL tryptone phosphate broth, all supplied by Sigma-Aldrich Co) and stored around −70 °C prior to analysis by EIV nucleoprotein quantitative reverse transcriptase polymerase chain reaction (EIV NP qRT-PCR) or embryonated hen’s egg titration [8]. EIV NP qRT-PCR measures the presence of genetic material. For the EIV NP qRT-PCR, automated RNA extraction was performed on 100 µL of swab sample using a KingFisher Flex system (Fisher Scientific, Loughborough, Leicestershire, UK). Results were expressed as number of EIV NP RNA copy per 2 µL of initial swab extract, positive results ≥100 or ≥2 if log transformed. The cut-off point (100) was determined by comparison with the EIV NP ELISA, which was used as the gold standard diagnostic method prior to the qRT-PCR assay development. The sensitivity and specificity of the qRT-PCR are 95.8% and 89.1%, respectively, with a cut-off threshold of 100 mRNA copies. The sensitivity of the assay has been favoured to maximise the surveillance for a relatively rare infection among diagnostic sample throughput. The specificity of the qRT-PCR is likely to be under-evaluated, which is not unusual when the new assay is superior to the gold standard method with which it is being compared [12]. Detection of EIV shedding by qRT-PCR is referred to in the OIE Terrestrial Manual EIV (2016), but no EIV qRT-PCR methods have been validated in line with the OIE validation template yet [8]. Based on previous method comparisons [12,14] and following the principles for the replacement, refinement and reduction (3Rs) of animals in research, only samples positive by EIV NP qRT-PCR (≥2 log NP mRNA copy/2 µL nasal swab extract) were titrated in embryonated hens’ eggs. The titration in embryonated hens’ eggs assesses the presence of live infectious virus. Results are expressed as log EID_50_/mL [12,22] of swab extract as previously described [26]. Only samples positive by EIV NP qRT-PCR (≥2 log NP mRNA copy/2 µL nasal swab extract) were titrated in embryonated hens’ eggs. 

### 2.6. Statistical Analysis

Statistical analyses were performed with STATGRAPHICS Centurion XVI, version 16.1.12 (StatPoint Technologies, Inc., Warrenton, VA, USA). The results of standardised skewness and standardised kurtosis tests were used to confirm normal distribution of the data and whether the results were distributed normally. Based on normal distribution (i.e., the results of the standardised skewness and standardised kurtosis tests), the Student’s *t*-test (S) or the Wilcoxon signed rank test (W) were used to compare the groups at specific time points. A Barnard’s test was used to compare the need for antibiotic treatment per group (online calculator: http://scistatcalc.blogspot.co.uk/2013/11/barnards-test-calculator.html). For each statistical test, the statistical power (S power) of the study, with a sample size of 5 controls and 7 vaccinates and a 95% confidence interval, was controlled with the DSS Research sample size and statistical power calculators (https://www.dssresearch.com/KnowledgeCenter/ToolkitCalculators/StatisticalPowerCalculators). If statistical power is high (e.g., ≥80%), the probability of making a Type II error (false negative) or concluding that there is no difference between the groups, when in fact there is one, decreases. A statistical power ≥80% is usually required for studies [27]. The level of significance was set as 0.05.

## 3. Results

### 3.1. Antibody Response

#### 3.1.1. SRH Antibody Response

**Figure 1:**
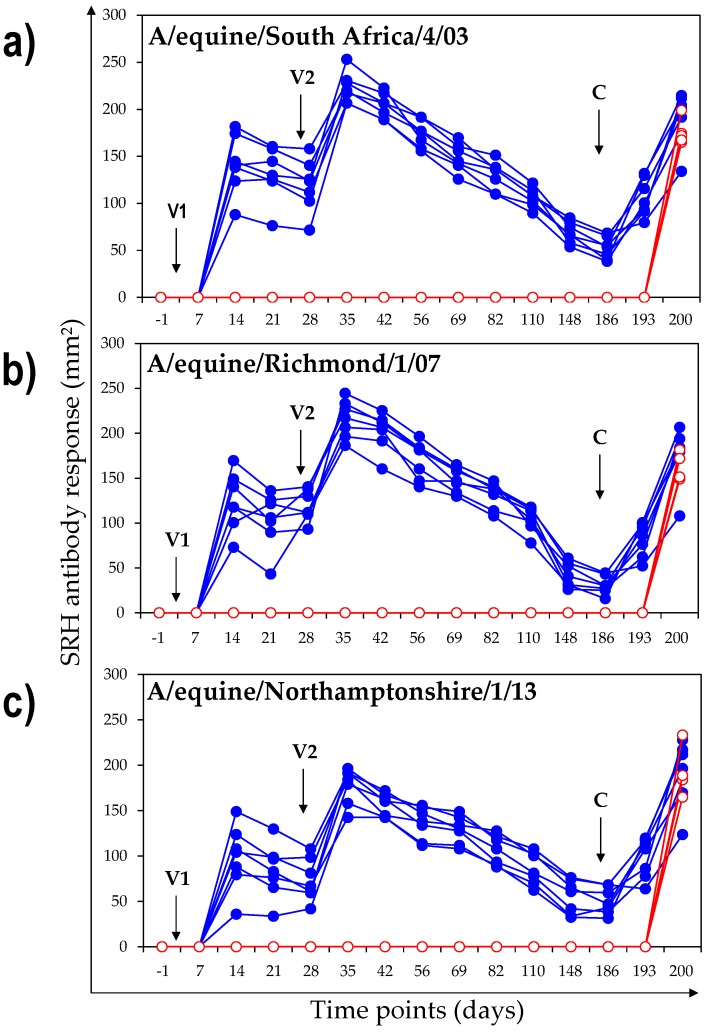
Individual single radial haemolysis (SRH) antibody response against A/equine/South Africa/4/03 (**a**) A/equine/Richmond/1/07 (**b**) and A/equine/Northamptonshire/1/13 (**c**). Vaccination and infection are indicated with arrows. Closed circles = vaccinated ponies; open circles = control ponies.

SRH antibody responses are presented in Figure 1. All vaccinated ponies responded to initial immunisation (V1) and developed a detectable SRH antibody response against the antigens tested from day 14 post V1. The intensity of the SRH antibody response varied from pony to pony and depending on the antigen used for the assay. After the second vaccination (V2 Day 28), the SRH antibody response was significantly increased in all vaccinated ponies when measured between days 28 and 35 (*p* value = 0.022 for A/equine/Richmond/1/07, paired samples signed rank test; 0.00004 for A/equine/South Africa/4/03, paired samples Student’s *t*-test, and 0.00002 for A/equine/Northamptonshire/1/13, paired samples Student’s *t*-test). The average (standard deviation) SRH antibody response 14 days after V2 (onset of immunity) was 223.1 ± 16.1 mm^2^ against A/equine/South Africa/4/03 (Day 35), 215.9 ± 20.7 mm^2^ against A/equine/Richmond/1/07 (Day 35) and 177.5.3 ± 20.0 mm^2^ against A/equine/Northamptonshire/1/13 (Day 35). SRH antibody titres declined in all ponies between V2 and Day 186 (day of infection). On Day 186, all vaccinated ponies showed detectable SRH antibody titres, with an average of 52.3 ± 11.8 mm^2^ against A/equine/South Africa/4/03, 30.9 ± 10.1 mm^2^ against A/equine/Richmond/1/07 and 50.8 ± 14.7 mm^2^ against A/equine/Northamptonshire/1/13.

All vaccinated ponies showed a statistically significant increase of SRH antibody titres against all EIV antigens between day 186 (day of infection) and day 193 (7 days after infection, anamnestic memory response of the antibody response stimulated by vaccination), with *p* values of 0.0015 for A/equine/Richmond/1/07, 0.0016 for A/equine/South Africa/4/03, and 0.006 for A/equine/Northamptonshire/1/13 (S). On Day 200 (14 days after infection), the average SRH antibody titres were 178.1 ± 32.4 mm^2^ against A/equine/Richmond/1/07, 190.2.1 ± 28.3 mm^2^ against A/equine/South Africa/4/03 and 190.1 ± 35.5 mm^2^ against A/equine/Northamptonshire/1/13.

None of the control ponies had measurable SRH antibody titres prior to experimental infection with A/equine/Northamptonshire/1/13. Overall, results from control ponies confirmed the absence of contact with naturally-circulating EIV during the study. Fourteen days after experimental infection with A/equine/Northamptonshire/1/13 (day 200), all of the control animals seroconverted to the H3N8 EIV HA antigens. The average SRH antibody titres were 175.4 ± 13.6 mm^2^ against A/equine/South Africa/4/03, 161.0 ± 14.8 mm^2^ against A/equine/Richmond/1/07 and 192.0 ± 25.1 mm^2^ against A/equine/Northamptonshire/1/13.

On day 200 (14 days after infection), the titres of SRH antibodies in the control and vaccinated groups were not statistically different, when tested against A/equine/South Africa/4/03 (*p* value = 0.31, S), A/equine/Richmond/1/07 (*p* value = 0.86, W) or against A/equine/Northamptonshire/1/13 (*p* value = 0.92, S).

#### 3.1.2. HI Antibody Response

**Figure 2:**
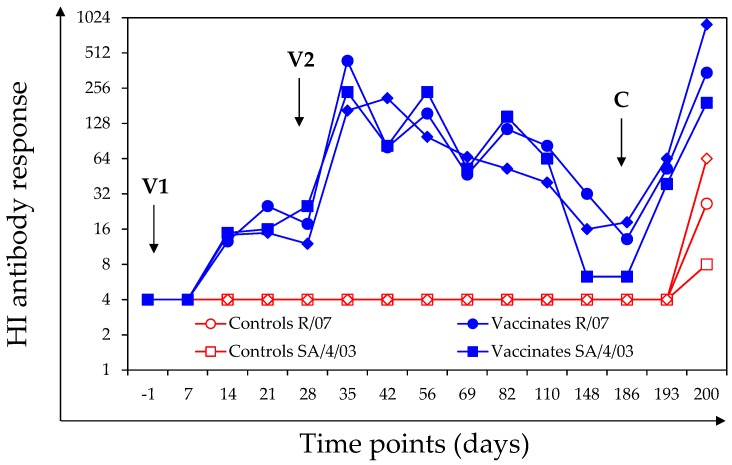
Group haemagglutination inhibition (HI) antibody response against A/equine/South Africa/4/03 (SA/4/03), A/equine/Richmond/1/07 (R/07) and A/equine/Northamptonshire/1/13 (N/1/13). Vaccination and infection are indicated with arrows. Closed symbols = vaccinated ponies; open symbols = control ponies. HI titres <8 were converted into 4, for graphical reasons.

Haemagglutination inhibition antibody titres against at least one antigen were detected in 6 out of 7 vaccinated ponies after V1. After the second vaccination (V2 day 28), the HI antibody response against at least one antigen was significantly increased in all of the vaccinated ponies (day 28 and day 35 comparison: *p* value = 0.002 for A/equine/South Africa/4/03, paired sample signed rank test; *p* value = 0.011 for A/equine/Richmond/1/07, paired Student’s *t*-test; *p* value = 0.003 for A/equine/Northamptonshire/1/13, paired Student’s *t*-test). The intensity of the HI antibody response varied from pony to pony and depending on the antigen used for the assay. When tested on day 186 (day of infection), 6 out of the 7 vaccinated ponies had measurable HI antibody titres against at least one antigen. There was a significant increase in the HI antibody titres (on Day 194 or Day 200, depending of the pony and antigen tested) in all of the vaccinated ponies after experimental infection with A/equine/Northamptonshire/1/13 (Figure 2).

There were no measurable HI titres in any of the control ponies during the vaccination phase of the study (day-1 to day 186). All of the control ponies seroconverted to at least one antigen after experimental infection with A/equine/Northamptonshire/1/13, but overall HI titres remained low. On day 200, the HI antibody titres against A/equine/Richmond/1/07 (*p* value = 0.0014, S), A/equine/South Africa/4/03 (*p* value = 0.0006, S) or A/equine/Northamptonshire/1/13 (*p* value = 0.004, W) in the vaccinated group were statistically significantly higher than in the control group. 

#### 3.1.3. TT Antibody Response

Tetanus toxoid (TT) antibody titres are presented in Table 2. There were no TT antibody titres on day-1. Tetanus antibody antibody titres were measured in all of the vaccinated ponies on Day 56 (52.1 ± 21.0 IU/mL) but none of the unvaccinated control ponies had TT antibody titres. All of the ponies enrolled in the study were administered anti-tetanus serum administration on day 61 or day 62 (castration of male ponies). On day 69 (around one week after anti-tetanus serum administration), TT antibody titres were measured in all of the ponies (25.7 ± 11.3 IU/mL and 0.33 ± 0.15 IU/mL in the vaccinated and control groups, respectively). On day 186 (day of infection), TT antibody titres were measured in all of the vaccinated ponies (3.2 ± 1.2 IU/mL) but not in control ponies. 

### 3.2. Clinical Signs of Disease after Experimental Infection with A/Equine/Northamptonshire/1/13

**Figure 3:**
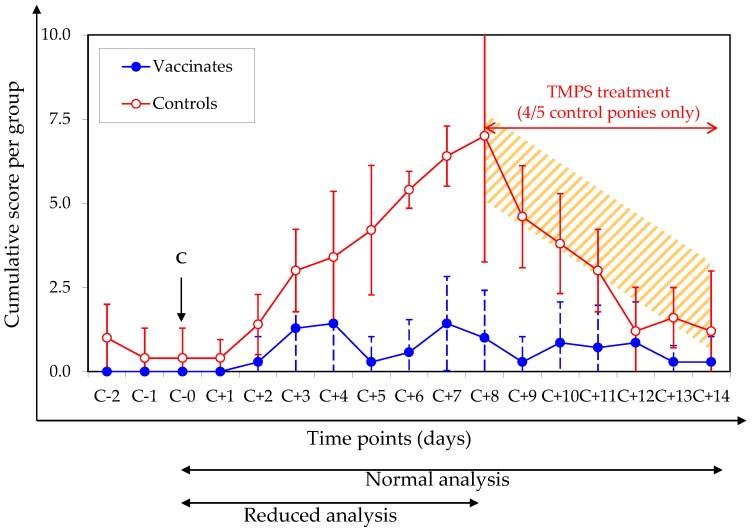
Cumulative clinical signs of disease. The day of infection (C; Day 186 = C-0) is indicated with an arrow. Closed circles = vaccinated ponies; open circles = control ponies. The period of antibiotic treatment (trimethoprim/sulfadiazine, TMPS) is represented as a shaded area. The time periods considered for the normal and the reduced analyses are also indicated.

Vaccination significantly reduced the clinical signs of disease induced 158 days (approximately 22.5 weeks) after the second immunisation by an experimental infection with the EIV strain A/eq/Northamptonshire/1/13. The vaccinated group had a significantly lower cumulative clinical score and average clinical score per day. Pyrexia was recorded in 4 out of 5 control animals for 2 to 4 days and 1 out of 7 vaccinates for 1 day. Significant differences were also observed for nasal discharge and coughing, with lower occurrence and severity in the vaccinated animals than in the controls. There was no difference in ocular discharge scores between the control and vaccinated groups. The results are reported in Table 3 and Figure 3.

Four out of five control ponies received antibiotic treatment on day 194 (8 days after experimental EIV infection) in order to control secondary bacterial infection and to improve recovery (trimethoprim/sulfadiazine, for 7 days). None of the vaccinated ponies required antibiotic treatment. There was a statistically significant difference in the number of animals treated with antibiotics per group (*p* value = 0.000482; Barnard’s test). In order to take into account the potential effect of antibiotic treatment on the clinical signs of disease, a reduced statistical analysis from the day of infection (day 186) up to the first day of treatment (day 8 after infection) was also performed. Results are presented in Table 3. The statistical power of the study was sufficiently high (≥80%) for all parameters except the average of the severity score for nasal discharge per day and the average of the cumulative score for ocular discharge due to individual variability within a group.

### 3.3. Virus Shedding after Experimental Infection with A/Equine/Northamptonshire/1/13

**Figure 4:**
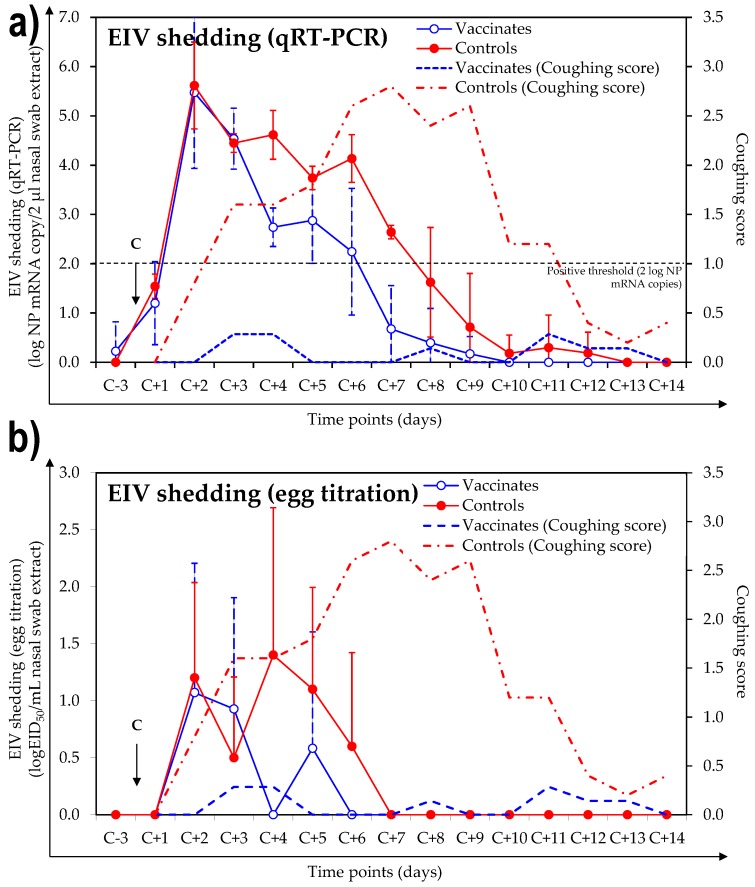
Virus shedding measured by equine influenza virus (EIV) NP qRT-PCR (**a**) or embryonated hens’ eggs titration (**b**) and coughing score. The day of infection (C; day 186 = C-0) is indicated with an arrow. Closed circles = EIV shedding measured in NS from vaccinated ponies; open circles = EIV shedding measured in NS from control ponies; dotted line = coughing score for vaccinated ponies; broken line = coughing score for control ponies. The dotted line (**a**) represents the threshold above which a sample is considered positive for EIV by qRT-PCR. Only samples which tested positive by EIV NP qRT-PCR (≥2 log NP mRNA copy/2 µL nasal swab extract) were titrated in embryonated hens’ eggs. Other samples (<2 log NP mRNA copy/2 µL nasal swab extract) were considered negative and represented as such (**b**).

All of the control and vaccinated ponies were positive for EIV shedding after experimental infection with A/equine/Northamptonshire/1/13 when measured by EIV NP qRT-PCR. The EIV NP mRNA copy number/2 µL of nasal swab extract and the number of days positive are presented in Supplementary Materials Table S2. Results for days 183 (3 days before infection) to 200 (14 days after infection/challenge) are illustrated in Figure 4. Control ponies shed EIV from day 188 (2 days before infection/challenge) to day 195 (9 days after infection), with an average duration of 6.6 ± 0.9 days. EIV shedding was measured in vaccinated ponies from day 187 (1 day after infection) to day 193 (7 days after infection), with an average duration of 4.7 ± 1.01 days. All of the control ponies and 5 out of 7 vaccinated ponies were positive for EIV shedding after experimental infection with A/equine/Northamptonshire/1/13 when titrated in embryonated hen’ eggs. Control ponies shed infectious EIV from day 188 (2 days after infection) to day 192 (6 days after infection), with an average duration of 3.2 ± 1.8 days. Infectious EIV shedding was measured in vaccinated ponies from day 188 (2 days after infection) to day 191 (5 days after infection), with an average duration of 1.4 ± 0.8 days. The duration of EIV shedding (as measured by qRT-PCR or titrated in embryonated hens’ eggs) was significantly shorter in vaccinated ponies than in control ponies (*p* = 0.006 and *p* = 0.04, respectively, S, Supplementary Materials Table S2). The statistical power of the study was sufficiently high (≥80%) for all parameters except the average titre per day of EIV shedding (qtPCR and embryonated hens’ eggs) average of the cumulative shedding of EIV (embryonated hens’eggs) and average duration of EIV shedding (embryonated hens’eggs) due to individual variability within a group. 

## 4. Discussion

The vaccine efficacy study reported here was specifically designed to test the protection of horses induced by a commercially available EI vaccine under unfavourable conditions. For this purpose, two major elements were taken into consideration: (1) the timing of the experimental infection (i.e., infection 158 days after second immunisation (V2)) and (2) the nature of the EIV strain used for this purpose (i.e., recent FC2 EIV strain) in relation to the EI vaccine strains (i.e., FC1 EIV and European lineage strains). A previous study has shown that ponies vaccinated with an ISCOM-based EI vaccine and experimentally infected with EIV only 78 days after V2 were significantly protected against clinical signs of equine influenza but virus shedding, while significantly reduced, was still sufficient to effectively transmit EI to sentinel ponies for up to 6–8 days after experimental infection [14]. 

Virus excretion was measured using both detection of mRNA (qRT-PCR) and infectious EI (titration in embryonated hens’ eggs). This design could have been strengthened further using sentinel naïve ponies to determine if the amounts of infectious virus excreted by vaccinated ponies were sufficient to actively transmit EI, as shown previously [14]. Antibody titres were measured using HI and SRH assays in the present study. While the former is based only haemagglutinin, SRH (a complement fixing assay) targets both haemagglutinin and neuraminidase. Neuraminidase plays an increasingly recognised role as an influenza immunogen, through reducing the amount of virus released from infected cells [28]. In humans, antibodies to both haemagglutinin and neuraminidase can help to predict immunity to influenza and reduce influenza-associated disease [29]. It remains to be demonstrated whether antibodies to neuraminidase also play a key role in protection against influenza in horses [28,29].

The EI vaccine used in this study (i.e., Equilis Prequenza-Te) contains whole EIV strains A/equine/South Africa/4/03 (H3N8, American lineage, Florida Clade 1 sublineage, isolated in 2003) and A/equine/Newmarket/2/93 (H3N8, European lineage, isolated in 1993). This vaccine is considered to be partially updated in relation to the most recent OIE ESP recommendation on EI vaccine strain composition that supports the inclusion of representative EIV strains from both FC1 and FC2 sublineages [30]. The challenge strain A/equine/Northamptonshire/1/13 used in this study is a recent FC2 strain (isolated in 2013) that presents significant differences in the nucleotide sequence encoding HA from the FC1 EIV vaccine strain A/equine/South Africa/4/03 [7] (Supplementary Materials Figure S1). Antigenic differences in HA between circulating, vaccine and reference EIV strains are usually evaluated using the HI assay in combination with a panel of EIV-specific ferret antisera, supported by antigenic cartography of HI data and genetic sequencing of the haemagglutinin 1 (HA1) gene. The HI titres in ferrets against A/equine/Northamptonshire/1/13 were 5.7 to 8-fold lower than those raised against A/equine/South Africa/4/03 (vaccine strain) and comparable to HI titres obtained with ferret anti-sera against A/equine/Richmond/1/07 or the more recent A/equine/Devonshire/1/11 (closely related to A/equine/Northamptonshire/1/13), respectively [7]. Such differences in HI titres are related to the antigenic diversity in the HA sequence between FC2 strains such as A/equine/Northamptonshire/1/13 and FC1 strains, such as A/equine/South Africa/4/03. These antigenic differences form the basis for the OIE ESP recommendations to include both FC1 and FC2 EIV strains in EI vaccines.

The immunogenicity of EI vaccines is based on the type and amount of antigen and the adjuvant, which enhances and modulates the immune response and targets antigen to antigen-presenting cells, thus stimulating both humoral and cell-mediated immunity. This helps to explain why EI vaccines induce far higher and serotypically broader protection than the vaccines used to immunize humans against seasonal influenza. This may also perhaps explain why confirmed vaccination breakdown—the sudden appearance of disease in an individual or herd in which immunity had previously appeared or been assumed to be adequate—is relatively infrequent in horses that are regularly and appropriately vaccinated against EIV [6,24]. Protecting the equine population against EIV is complex. There can be considerable individual variation in response to vaccination during field use, depending on a number of factors, including the innate immune system but also age and stress (training, diet changes, transport, etc.) [31]. Immunisation is at its most effective when a high level of the population is covered.

The experimental infection in the present study was performed 158 days (around 5.2 months) after V2, at a time when the first revaccination (V3) is recommended (subsequent revaccinations would usually take place every 12 months). This specific timing was selected to maximise the susceptibility of vaccinated ponies to EIV infection (i.e., immunity gap). Under these conditions (i.e., combination of EIV strain heterogeneity and immunity gap), immune responses induced by EI vaccination was placed under significant pressure and tested using the natural host model commonly used to evaluate EI vaccine efficacy.

Clinical signs of disease were significantly reduced in vaccinated ponies when compared with unvaccinated control ponies, despite SRH antibody titres being below the threshold proposed for protection against clinical signs of disease induced by experimental infection (i.e., 85 mm^2^ against homologous EIV strain) [9,10]. There was a notable reduction in pyrexia, which confirms the animal welfare benefits of vaccination. The dramatic reduction in coughing in the vaccinated ponies compared to the controls is likely to help to reduce the risk of EIV dissemination and disease transmission. Nasal discharge usually evolves after experimental infection with EIV, with an increase in magnitude (from mild to copious discharge) and nature (from serous discharge in the first few days after viral infection becoming mucopurulent due to the establishment secondary bacterial infection). While this clinical marker was less affected by vaccination, there was a statistically significant reduction in the need to use antibiotics to prevent and/or control secondary bacterial infection in the vaccinated group compared to the controls. A canarypox-vectored EI vaccine and ISCOMatrix-adjuvanted EI vaccines have been previously shown to protect against the EIV strains A/equine/Sussex/89, /Kentucky/91 and /Kentucky/9/95, during the immunity gap (experimental infection 5–6 months after V2) [32,33,34]. However, heterogeneity between vaccine and challenge strains was limited in these studies. Despite the significant time between V2 and the experimental infection (i.e., 158 days), the clinical outcome measured in the vaccinated group in the present study was similar to the outcome measured in ponies vaccinated with (1) an EI vaccine based on a canarypox-vectored vaccine expressing the haemagglutinin gene of EIV and experimentally infected with EIV 14 days after the second immunisation [35] and (2) a whole inactivated EI vaccine as well as experimental infection with EIV 14 or 15 days after V2 [12,26]. The clinical protection achieved in the current study was also similar or superior to the one recorded in ponies vaccinated with an ISCOM-based EI vaccine and experimentally infected with EIV 14 days [25] or 78 days after V2 [14], respectively. 

There was little or no difference in the amount of EIV shed measured between control and vaccinated ponies during the first few days after experimental infection. This is likely to be a consequence of the detectable but limited amount of EIV HA-specific antibodies measured at the time of infection, being high enough to provide clinical protection but insufficient to completely prevent EIV infection. Previous studies have clearly illustrated a correlation between SRH antibody titres and protection [9,10] but high levels of SRH antibody are necessary to provide sterilising immunity and prevent EIV infection (sterilising immunity is usually characterised by an absence of clinical signs of disease, virus shedding and seroconversion subsequent to experimental infection). Sterilising immunity can be observed shortly after V2. While it has not been demonstrated experimentally, sterilising immunity may also occur at peaks of immunity induced by subsequent boost immunisations (V3, V4, etc.) [35]. It is important to note that the infectious dose was delivered by individual nebulisation, which significantly increases the amount of virus inhaled by ponies when compared with the historical room nebulisation procedure previously used in numerous EI vaccine studies. Unvaccinated and unprotected control ponies also shed significant amount of EIV and could be considered as a secondary source of infection (i.e., during the infectious phase of the study, vaccinated and control ponies were commingled with at least 2 control ponies in each room). Under these conditions, low levels of immunity on day 186 may have been overcome, resulting in no or little difference in virus shedding between vaccinated and control ponies immediately after experimental infection. However, EIV shedding quickly subsided in the vaccinated ponies when compared to the control animals. This observation supports a rapid mobilisation and anamnestic response of EIV-specific immunity induced by vaccination, as early as 4 days after infection, confirmed by seroconversion measured 7 days after experimental infection in the vaccinated ponies. This result clearly highlights the benefit of EI vaccination. Such an effect is usually not measurable in clinical trials with experimental infection set up at the onset of immunity conferred by vaccination (i.e., experimental infection 2 to 3 weeks after primary immunisation (V1 and V2)). Under such conditions, antibody titres present at the time of experimental infection are usually high enough to significantly limit initial EIV infection, which immediately impacts and reduces the amount of virus shed and detected [12,25,26,35].

Prior to V1, no antibodies against tetanus were detected. Unvaccinated control ponies remained negative throughout the study, except one week following tetanus antiserum administration when the titre was 0.33 ± 0.15 IU/mL. In vaccinated ponies, tetanus antibody titres were 52.1 ± 21.0 IU/mL one month following V2, 25.7 ± 11.3 IU/mL around 6 weeks after V2 (one week following tetanus antiserum administration) and 3.2 ± 1.2 IU/mL around 22.5 weeks (158 days) after V2. This is similar to the tetanus antibody titres measured in a previous study with an older version of this vaccine [36]. In that study, tetanus antibody titres were over 20 IU/mL 2 weeks after V2 and around 2 IU/mL at 22 weeks after V2 followed by lower levels (0.31 IU/mL) that were maintained for at least until 76 weeks after V2 and V3 has been shown to result in even higher tetanus antibody titres in horses that provide protection until the time of the next recommended annual revaccination [36]. These titres are far above those required for clinical protection (ToBI titre of 0.02 IU/mL)[37]. In a study in client-owned horses, tetanus antibody titres (ToBI) remained above 0.04 IU/mL (limit of detection of assay) for 12 months (25/26 horses), 24 months (16/16) or 36 months (8/8) after V3 [38]. These studies support protection of horses against tetanus for at least 17 months after the primary vaccination course and 24 months after the first revaccination with this vaccine. Based on the rather slow decrease of ToBi titers, it is clear that the protective titer may last even longer than observed in these studies [36]. As a consequence of the 24-month duration of immunity against tetanus, an alternating annual vaccination schedule consisting of an EI vaccine and a combined EI and tetanus vaccine is proposed after V1, V2 and V3 of a combined EI and tetanus vaccine [36].

In the event of an outbreak, and if there is an imminent risk of contact with EIV, boost EI vaccination may need to be considered, especially for horses where the immunisation schedule has been started recently [39]. While a shortening of the interval between V1 and V2 has been tested [25,40,41], an attempt to reduce the gap between V2 and V3 has been shown to be counter-productive, with a long term detrimental effect on humoral immunity induced by V3 and V4 boost immunisations [17]. This may mean that with current EI vaccine technology it is not possible to close the immunity gap and certainly reducing the interval between V2 and V3 is not recommended.

## 5. Conclusions

In the present study, the whole inactivated EI and tetanus vaccine (Equilis Prequenza Te) provided good clinical protection against a recent EIV strain, even when challenged 158 days after V2. All of the vaccinates had detectable but low SRH antibody titres at the time of experimental infection. The clinical protection demonstrated in the present study was similar to what has been observed for other EI vaccines when tested much sooner after immunisation, such as at the onset of immunity [12,25,32,42,43] or 2.5 months after V2 [14]. Virus shedding was measured after individual challenge by nebulisation in the present study, but the overall duration was significantly reduced in the vaccinates compared to the unvaccinated control ponies. This study illustrates that ponies immunised with this EI and tetanus vaccine (Equilis Prequenza Te) show limited evidence of the immunity gap when experimentally infected 158 days (around 5.2 months) months after V2 with a recent heterologous strain, which is representative of the EIV strains circulating in the EU.

## Figures and Tables

**Table 1:** Study design. D186 = time of experimental infection. n = number of animals; N/1/13 = A/equine/Northamptonshire/1/13; V = vaccination; Inf. C = experimental infection; C + 7 = 7 days after infection; C + 14 = 14 days after infection.

Group	n	Treatment	D0 (V1)	D28 (V2)	D186 ^1^
Controls	5	Placebo	PBS	PBS	N/1/13
Vaccinates	7	Vaccination	EI-TT vaccine	EI-TT vaccine	N/1/13
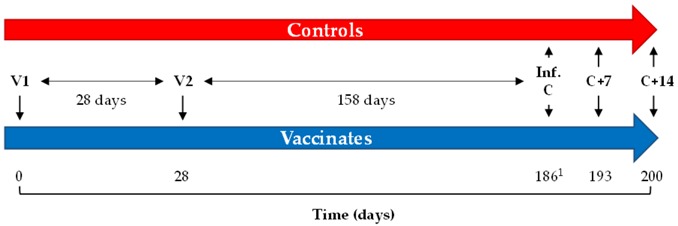					

^1^ D186: infection by individual nebulization.

**Table 2:** Tetanus toxoid antibody titres, expressed as international units (IU)/mL.

Pony #	Group	Day-1	Day 56	Day 69	Day 186 *
1	Vaccinates	0.0	57.31	42.15	4.43
2	Vaccinates	0.0	76.13	38.39	4.75
3	Vaccinates	0.0	51.00	16.14	2.69
4	Vaccinates	0.0	60.19	24.34	2.30
5	Vaccinates	0.0	44.42	13.35	3.85
6	Vaccinates	0.0	65.32	28.63	1.36
7	Vaccinates	0.0	10.30	16.78	3.01
8	Controls	0.0	0.0	0.58	0.0
9	Controls	0.0	0.0	0.36	0.0
10	Controls	0.0	0.0	0.18	0.0
11	Controls	0.0	0.0	0.27	0.0
12	Controls	0.0	0.0	0.28	0.0
average	Vaccinates	0.0	52.1	25.7	3.2
STDV	Vaccinates	0.0	21.0	11.3	1.2
average	Controls	0.0	0.0	0.33	0.0
STDV	Controls	0.0	0.0	0.15	0.0

* Day 186: day of infection by individual nebulisation.

**Table 3:** Clinical signs (mean ± SD) of disease after experimental infection with A/equine/Northamptonshire/1/13 and statistical analyses (vaccinated group versus control group). Significant differences are in bold text. Reduction (%, SD) = the reduction in clinical signs in the vaccinated group compared to the control group, A = analysis, S power = statistical power. * Student’s *t*-test; ** Wilcoxon’s test; *** Barnard’s test.

Clinical Sign	Controls	Vaccinates	Normal A ^1^	Reduced A ^2^	S Power
Cumulative clinical score	47.0 ± 9.8	9.6 ± 5.1	0.000006 *	0.00006 *	100%
Clinical severity score per day	3.9 ± 0.5	2.0 ± 0.9	0.006 **	0.004 *	99.9%
Disease duration (days)	12.0 ± 2.0	4.1 ± 2.2	0.00009 *	0.004 **	100%
Pyrexia duration (days)	1.0 ± 0.7	0.1 ± 0.4	0.033 **	0.033 **	82.7%
Frequency of pyretic ponies	4/5 (80%)	1/7 (14.3%)	0.032 ***	na	na
Cumulative nasal discharge score	25.6 ± 5.9	7.9 ± 4.2	0.0001 *	0.005 **	100%
Nasal discharge severity score per day	2.5 ± 0.1	2.0 ± 1.0	0.14 *	0.04 **	37%
Nasal discharge duration (days)	10.2 ± 2.8	3.4 ± 1.9	0.0005 *	0.001 *	99.9%
Cumulative cough score	19.6 ± 6.8	1.3 ± 3.0	0.005 **	0.004 **	100%
Cough severity score	1.3 ± 0.5	0.1 ± 0.2	0.005 **	0.005 **	100%
Cough duration (days)	8.8 ± 2.2	0.9 ± 1.9	0.004 **	0.00003 *	100%
Cumulative ocular discharge score	0.6 ± 0.9	0.4 ± 0.8	0.73 *	0.45 *	10.6%

^1^ From the day of infection and up to day 14 after infection. ^2^ from the day of infection and up to the first day of antibiotic treatment.

## Supplementary Materials

The following are available online at http://www.mdpi.com/2076-393X/6/3/38/s1, Figure S1: EIV phylogenetic tree, Figure S2: CONSORT flow chart, Table S1: CONSORT check list, Table S2: Virus shedding after experimental infection.
