# Supplementary material for: The Immunity Gap Challenge: Protection against a Recent Florida Clade 2 Equine Influenza Strain
Source: Vaccines (Basel). 2018 Jul 2;6(3):38. doi: 10.3390/vaccines6030038 (PMC6161116; doi:10.3390/vaccines6030038)
Supplement: Supplementary file 1 [file vaccines-06-00038-s001.zip › Paillot et al EIV Immunity Gap 2017 Supplementary Figure JUN18.pdf]

The Immunity Gap challenge: protection against a recent Florida Clade 2 equine influenza strain. SUPPLEMENTARY FIGURE 1

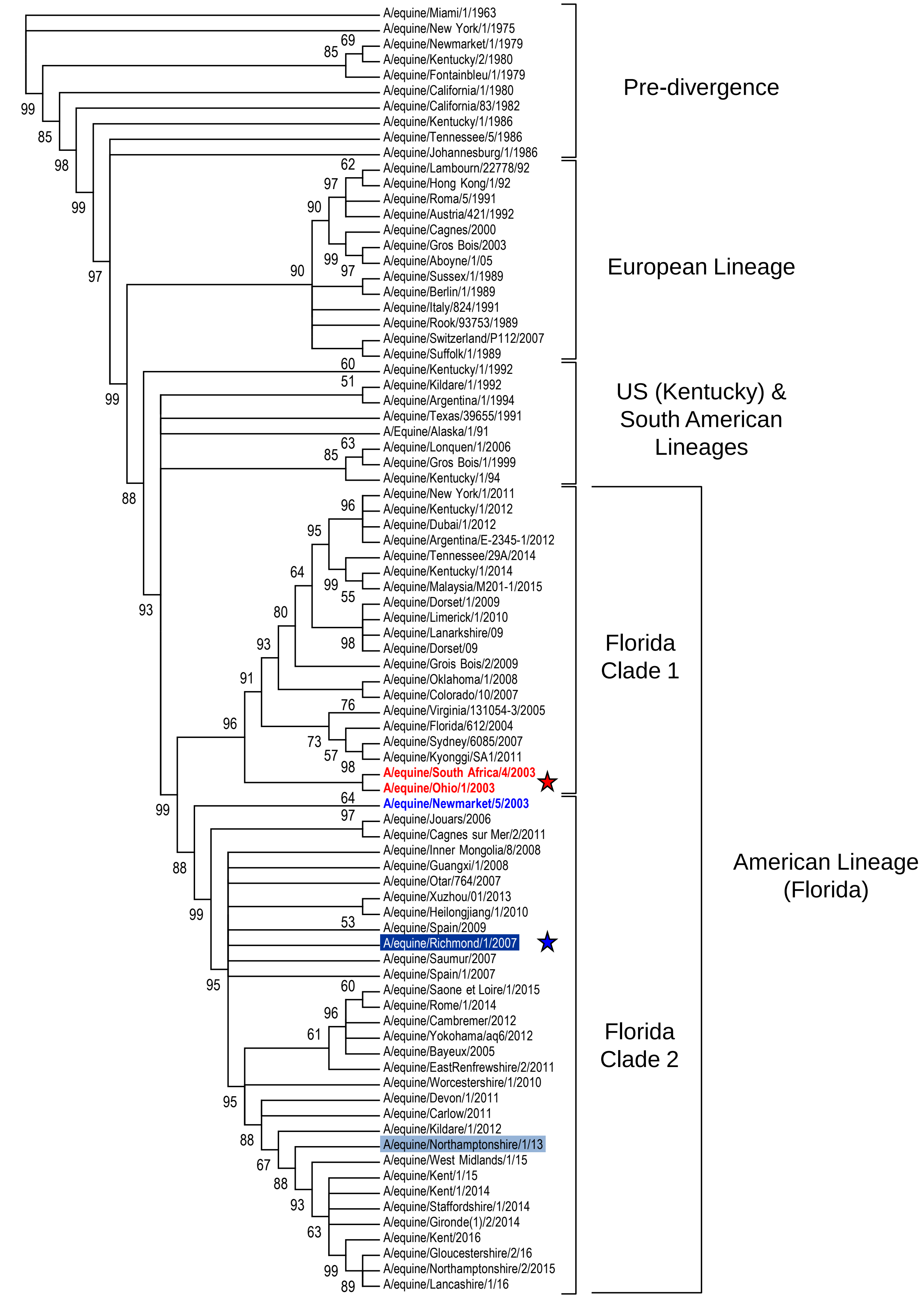

Supplementary Figure 1 (Legend): Phylogenetic analysis of the HA1 nucleotide sequence for 83 EIV strains, with a focus on FC2 EIV strains (GISAID EpiFlu™ database). The FC1 and FC2 prototype EIV strains are indicated in red and blue bold text, respectively. The FC1 and FC2 representative EIV strains recommended by the OIE ESP for inclusion in EI vaccine are indicated with a red or blue star, respectively. The EIV strain used as the challenge strains are in the blue coloured boxe. Neighbor-Joining tree, Test phylogeny: Boostrap method with 1000 replications, Method: Maximum composite Likelihood. MEGA6 (Tamura et al., 2013).

The Immunity Gap challenge: protection against a recent Florida Clade 2 equine influenza strain. **SUPPLEMENTARY TABLE 2**

Supplementary Table 2. Virus shedding after experimental infection with A/equine/Northamptonshire/1/13 and statistical analyses (vaccinated group versus control group). S power = statistical power. Significant differences are in bold text. [1] Student's T-test; [2] Wilcoxon's test.

**Virus shedding (EIV qRT-PCR)**	**Controls**	**Vaccinates**	**p-value**	**S. power**
Average cumulative EIV shedding (log NP mRNA copy/2 µl) [1]	29.7±3.0	20.3±4.7	**0.0028**	99.5%
Average EIV titre per positive day of shedding (log NP mRNA copy/2 µl) [1]	4.5±0.3	4.4±1.2	0.86	7.6%
Average EIV shedding duration (days) [1]	6.6±0.9	4.7±1.0	**0.006**	96.4%
**Virus shedding (embryonated hen's eggs titration)**	**Controls**	**Vaccinates**	**p-value**	
Average cumulative EIV shedding ($logEID_{50}/mL$)[2]	4.8±3.7	2.5±1.9	0.28	35.6%
Average EIV titre per positive day of shedding ($logEID_{50}/mL$) [2]	1.4±0.4	1.7±0.7	0.35	24.0%
Average EIV shedding duration (days) [1]	3.2±1.8	1.4±0.8	**0.04**	67.3%
