# Supplementary material for: The Immunity Gap Challenge: Protection against a Recent Florida Clade 2 Equine Influenza Strain
Source: Vaccines (Basel). 2018 Jul 2;6(3):38. doi: 10.3390/vaccines6030038 (PMC6161116; doi:10.3390/vaccines6030038)

CONSORT

TRANSPARENT REPORTING of TRIALS

CONSORT 2010 Flow Diagram (The Immunity Gap challenge: protection against a recent Florida Clade 2 equine influenza strain).

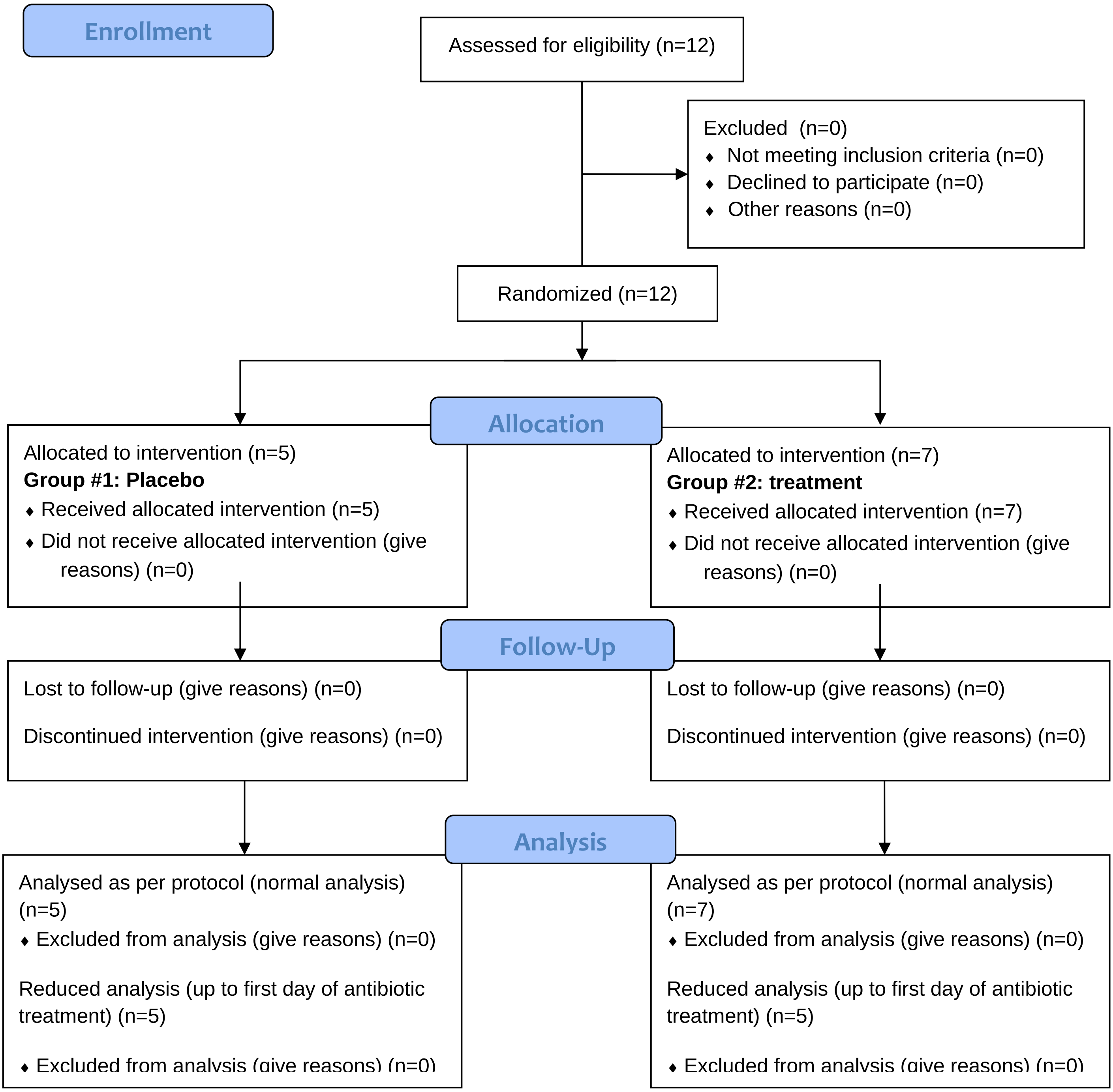

Supplement: Supplementary file 1 [file vaccines-06-00038-s001.zip › Paillot et al EIV Immunity Gap CONSORT 2017 Flow Diagram.pdf]
