# Supplementary material for: The Immunity Gap Challenge: Protection against a Recent Florida Clade 2 Equine Influenza Strain
Source: Vaccines (Basel). 2018 Jul 2;6(3):38. doi: 10.3390/vaccines6030038 (PMC6161116; doi:10.3390/vaccines6030038)
Supplement: Supplementary file 1 [file vaccines-06-00038-s001.zip › Paillot et al EIV Immunity Gap CONSORT check list.pdf]

CONSORT 2010 checklist of information to include when reporting a randomised trial*

The Immunity Gap challenge: protection against a recent Florida Clade 2 equine influenza strain.

Section/Topic	Item No	Checklist item	Reported on page No
**Title and abstract**			
	1a	Identification as a randomised trial in the title	No
	1b	Structured summary of trial design, methods, results, and conclusions (for specific guidance see CONSORT for abstracts)	Yes
**Introduction**			
Background and objectives	2a	Scientific background and explanation of rationale	Yes
	2b	Specific objectives or hypotheses	Yes
**Methods**			
Trial design	3a	Description of trial design (such as parallel, factorial) including allocation ratio	No
	3b	Important changes to methods after trial commencement (such as eligibility criteria), with reasons	None reported
Participants	4a	Eligibility criteria for participants	Mat & meth s2.1
	4b	Settings and locations where the data were collected	Mat & meth s2.1
Interventions	5	The interventions for each group with sufficient details to allow replication, including how and when they were actually administered	Mat & meth s2.1
Outcomes	6a	Completely defined pre-specified primary and secondary outcome measures, including how and when they were assessed	Mat & meth s2.3 and s2.4
	6b	Any changes to trial outcomes after the trial commenced, with reasons	No
Sample size	7a	How sample size was determined	Mat & meth s2.1
	7b	When applicable, explanation of any interim analyses and stopping guidelines	Not applicable
Randomisation:			
Sequence generation	8a	Method used to generate the random allocation sequence	Mat & meth s2.1
	8b	Type of randomisation; details of any restriction (such as blocking and block size)	No
Allocation concealment mechanism	9	Mechanism used to implement the random allocation sequence (such as sequentially numbered containers), describing any steps taken to conceal the sequence until interventions were assigned	Mat & meth s2.1

Implementation	10	Who generated the random allocation sequence, who enrolled participants, and who assigned participants to interventions	No
Blinding	11a	If done, who was blinded after assignment to interventions (for example, participants, care providers, those assessing outcomes) and how	Mat & meth s2.1
	11b	If relevant, description of the similarity of interventions	Not applicable
Statistical methods	12a	Statistical methods used to compare groups for primary and secondary outcomes	Mat & meth s2.6
	12b	Methods for additional analyses, such as subgroup analyses and adjusted analyses	Not applicable
**Results**			
Participant flow (a diagram is strongly recommended)	13a	For each group, the numbers of participants who were randomly assigned, received intended treatment, and were analysed for the primary outcome	Table 1 and CONSORT flow chart
	13b	For each group, losses and exclusions after randomisation, together with reasons	Not applicable
Recruitment	14a	Dates defining the periods of recruitment and follow-up	Not applicable
	14b	Why the trial ended or was stopped	Not applicable
Baseline data	15	A table showing baseline demographic and clinical characteristics for each group	Pre-vaccination characteristics are reported in results sections
Numbers analysed	16	For each group, number of participants (denominator) included in each analysis and whether the analysis was by original assigned groups	Table 1
Outcomes and estimation	17a	For each primary and secondary outcome, results for each group, and the estimated effect size and its precision (such as 95% confidence interval)	Results
	17b	For binary outcomes, presentation of both absolute and relative effect sizes is recommended	Not applicable
Ancillary analyses	18	Results of any other analyses performed, including subgroup analyses and adjusted analyses, distinguishing pre-specified from exploratory	Results s3.1.3
Harms	19	All important harms or unintended effects in each group (for specific guidance see CONSORT for harms)	Harms related to the experimental infection with EI, cf Results
**Discussion**			
Limitations	20	Trial limitations, addressing sources of potential bias, imprecision, and, if relevant, multiplicity of analyses	Yes
Generalisability	21	Generalisability (external validity, applicability) of the trial findings	Yes
Interpretation	22	Interpretation consistent with results, balancing benefits and harms, and considering other relevant	Yes

		evidence	
**Other information**			
Registration	23	Registration number and name of trial registry	Not applicable
Protocol	24	Where the full trial protocol can be accessed, if available	No
Funding	25	Sources of funding and other support (such as supply of drugs), role of funders	Aknowledgement, Author contributions, Conflicts of Interest

*We strongly recommend reading this statement in conjunction with the CONSORT 2010 Explanation and Elaboration for important clarifications on all the items. If relevant, we also recommend reading CONSORT extensions for cluster randomised trials, non-inferiority and equivalence trials, non-pharmacological treatments, herbal interventions, and pragmatic trials. Additional extensions are forthcoming: for those and for up to date references relevant to this checklist, see www.consort-statement.org.
